# Clinicopathological characteristics and outcomes of gastrointestinal stromal tumors with high progranulin expression

**DOI:** 10.1371/journal.pone.0245153

**Published:** 2021-01-07

**Authors:** In-Gu Do, Kyung Uk Jung, Dong-Hoe Koo, Yun-Gyoo Lee, Sukjoong Oh, Kyungeun Kim, Dong-Hoon Kim, Jin Hee Sohn, Byung Ho Son, Sung Ryol Lee, Jun Ho Shin, Hyung Ook Kim, Hungdai Kim, Ho-Kyung Chun, Ginette Serrero, Chang Hak Yoo

**Affiliations:** 1 Department of Pathology, Kangbuk Samsung Hospital, Sungkyunkwan University School of Medicine, Seoul, Republic of Korea; 2 Department of Surgery, Kangbuk Samsung Hospital, Sungkyunkwan University School of Medicine, Seoul, Republic of Korea; 3 Division of Hematology/Oncology, Department of Internal Medicine, Kangbuk Samsung Hospital, Sungkyunkwan University School of Medicine, Seoul, Republic of Korea; 4 A&G Pharmaceutical Inc., Columbia, Maryland, United States of America; 5 Greenebaum Cancer Center, University of Maryland, Baltimore, Maryland, United States of America; Universita degli Studi della Campania Luigi Vanvitelli, ITALY

## Abstract

**Background & aims:**

Progranulin (PGRN) is known to promote tumorigenesis and proliferation of several types of cancer cells. However, little is known about the clinicopathological features of patients with gastrointestinal stromal tumors (GISTs) with regard to PGRN expression.

**Methods:**

A retrospective analysis was performed on patients with GISTs who underwent curative surgical resection between 2007 and 2017. PGRN expression was evaluated by immunohistochemical (IHC) analysis and semi-quantitatively categorized (no expression, 0; weak, 1+; moderate, 2+; strong, 3+). Tumors with a staining intensity of 2+ or 3+ were considered high PGRN expression.

**Results:**

Fifty-four patients were analyzed; 31 patients (57%) were male. The median age at surgery was 60 years (range, 33–79), and the most common primary site was the stomach (67%). Thirty-five patients (65%) had spindle histology; 42 patients (78%) were separated as a high-risk group according to the modified National Institutes of Health (NIH) classification. High PGRN-expressing tumors were observed in 27 patients (50%), had more epithelioid/mixed histology (68% vs. 32%; *p* = 0.046), and *KIT* exon 11 mutations (76% vs. 24%; *p* = 0.037). Patients with high PGRN-expressing tumors had a worse recurrence-free survival (RFS) (36% of 5-year RFS) compared to those with low PGRN-expressing tumors (96%; *p*<0.001). Multivariate analysis showed that high PGRN expression and old age (>60 years) were independent prognostic factors for poor RFS.

**Conclusions:**

High PGRN-expressing GISTs showed more epithelioid/mixed histology and *KIT* exon 11 mutations. PGRN overexpression was significantly associated with poor RFS in patients with GISTs who underwent curative resection.

## Introduction

Gastrointestinal stromal tumors (GISTs) are rare tumors, but the most common form of mesenchymal tumors that originate from the interstitial cells of Cajal in the gastrointestinal wall [1]. GISTs are characterized by primary activating mutations in *KIT* or platelet-derived growth factor alpha (*PDGFRA*) genes that result in constitutive activation of receptor tyrosine kinase activity [2]. The standard treatment for localized GISTs is macroscopically complete surgical resection. Since the introduction of molecular-targeted therapy, such as imatinib, the outcomes of advanced GISTs have remarkably improved [3].

Recently, the mutations in the progranulin (PGRN) gene has been known to be related with the onset of neurodegenerative diseases [4]. While, in the oncology field, the association between PGRN and several malignancies such as leukemia, breast cancer, thyroid cancer, or ovary cancer has been discovered [5]. PGRN is a secreted glycoprotein recognized as an adipokine involved in diet-induced obesity, insulin resistance, or tumor progression cascade, including proliferation, migration, and angiogenesis [6, 7]. Recently, upregulation of PGRN in colorectal cancer has been shown to be correlated with tumor proliferation and vascular endothelial growth factor A (VEGF-A) expression, as well as increased microvessel density [8]. These increased Ki-67 and VEGF-A expressions, which were mediated by both tumor necrosis factor receptor-2 (TNFR2)/protein kinase B (Akt) and extracellular signal-regulated kinase (ERK) signaling pathways, suggested that PGRN might represent a kind of new growth factors in gastrointestinal cancer.

However, little is currently known about PGRN expression in GISTs with regard to clinicopathological features or the prognostic role of PGRN overexpression in patients with GISTs who have undergone curative resection. Therefore, this study evaluated PGRN expression in patients with GISTs who underwent curative resection and determined its prognostic role.

## Materials and methods

### Patients

We retrospectively reviewed the medical records of patients with GISTs who underwent surgery at Kangbuk Samsung Hospital (Seoul, Korea) between March 2007 and March 2017, and evaluated their tumor tissues. Patients who met the following criteria were included in our analysis: ≥18 years old; histologically confirmed GIST; curative-intent surgery without residual lesions; no history of other malignancies; and tumor tissue available for immunohistochemical (IHC) examination of PGRN expression. The study was approved by the Institutional Review Board (IRB) of Kangbuk Samsung Hospital (KBSMC 2019-07-014). Our analysis was a retrospective design using fully anonymized data, so the IRB waived the requirement for informed consent.

### Immunohistochemical analysis

**Fig 1:**
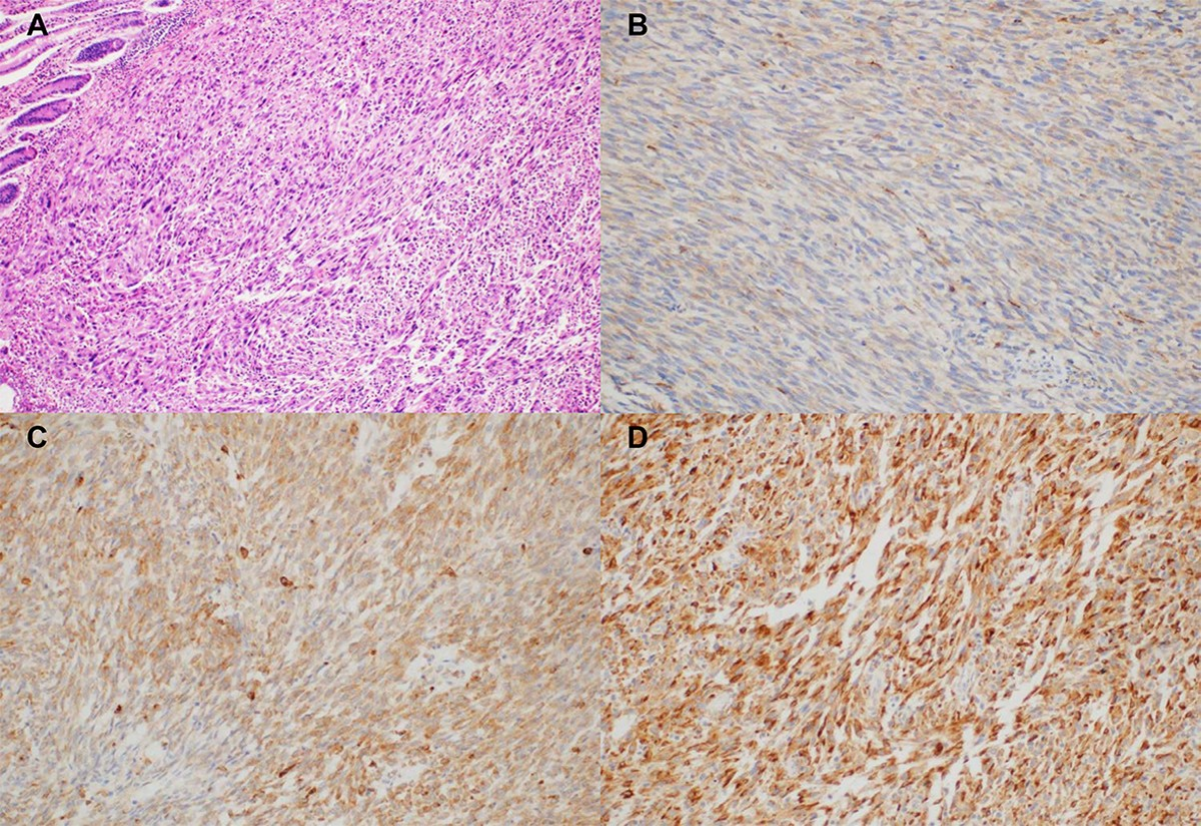
Progranulin expression in tumor cells by immunohistochemical staining (×200) (A) hematoxylin and eosin stain; (B) intensity 1+; (C) intensity 2+; (D) intensity 3+.

Specimens from formalin-fixed and paraffin-embedded samples at the time of surgery were collected for histological review and IHC analysis. IHC was performed using a Bond-max Autoimmunostainer (Leica Biosystems, Melbourne, Australia) with BondTM Polymer Refine Detection, DS9800 (Vision Biosystems, Melbourne, Australia) as described previously [9]. Briefly, formalin-fixed, paraffin-embedded, 4-μm-thick tissue sections were deparaffinized three times in xylene for a total of 15 min and subsequently rehydrated. Antigen retrieval was carried out at 97°C for 20 min in ER1 buffer. After blocking endogenous peroxidase activity with 3% hydrogen peroxidase for 10 min, slides were incubated with mouse monoclonal anti-progranulin antibody (PG359-7) (AG-20A-0052, Adipogen Life Sciences, Liestal, Switzerland) at a dilution of 1:500 for 15 min at room temperature. Negative controls (substitution of the primary antibody with Tris-buffered saline) were run simultaneously. The slides were assessed without knowledge of the clinical outcome. Progranulin was expressed in the cytoplasm of tumor or peritumoral cells with a granular pattern. PGRN expression in tumor cells was semi-quantitatively categorized, as previously described (no expression, 0; weak/focal, 1+; moderate/focal or diffuse, 2+; strong/diffuse, 3+) [10]. Tumor cells with ≥2+ PGRN staining were considered to be high PGRN-expressing tumors, while tumors with ≤1+ staining were considered to be low PGRN-expressing tumors (Fig 1).

### Statistical analysis

Associations between PGRN expression and clinical characteristics were assessed by univariate chi-square analysis using a dichotomized cut-off value for IHC positivity. Recurrence-free survival (RFS) was calculated as the duration from the date of surgery to the date of documented recurrence, death, or last follow-up (whichever occurred first). OS time was calculated as the duration from the date of surgery to the date of death or last follow-up. Survival curves were constructed using the Kaplan–Meier method and were compared using the log-rank test. Multivariate analysis was performed using the Cox proportional hazard regression model with the enter method. A two-sided P-value<0.05 was considered significant, and 95% confidence intervals (CIs) were calculated. All statistical analyses were performed using IBM SPSS ver. 25.0 (IBM Co., NY, USA).

## Results

### Baseline characteristics

**Fig 2:**
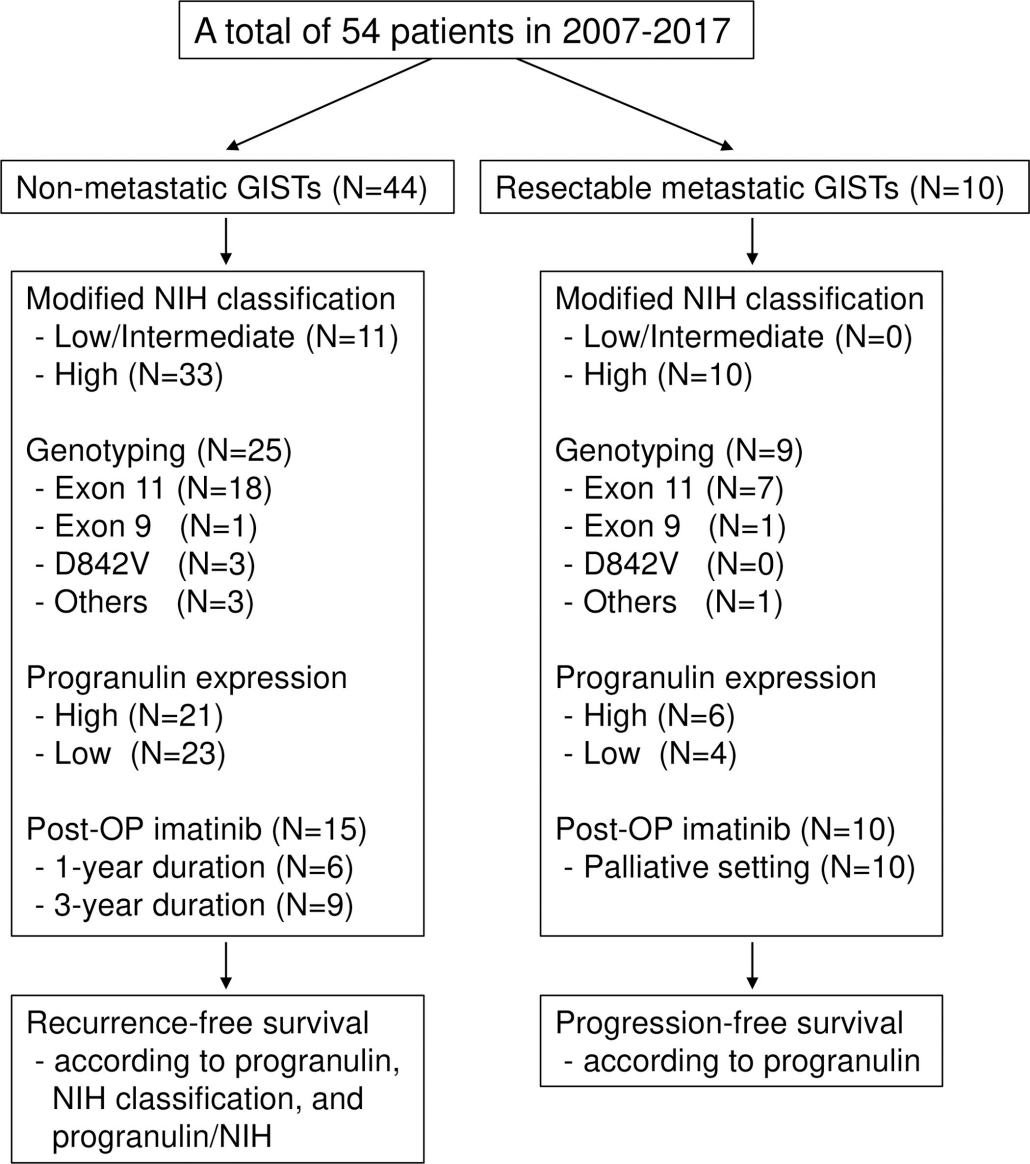
Flowchart of patients and clinicopathological features.

A total of 54 patients were evaluated and their clinical characteristics are summarized in Table 1. Thirty-one patients (57%) were male and the median age at the time of surgery was 60 years (range 33–79). Nine patients (17%) had diabetes mellitus, and the mean body weight and body mass index (BMI) was 62.2 kg (± standard deviation [SD], 8.7) and 23.7 m^2^ (± SD, 2.7), respectively. Forty-four patients (82%) had localized resectable disease, and 10 patients (19%) had resectable metastatic disease. The most common primary site was the stomach (67%), followed by the small bowel (26%). The common GIST histology was spindle cell type (65%), and the median tumor size was 8 cm (range, 2–47). Using the modified National Institutes of Health (NIH) classification and according to the size and mitosis index of primary tumors, the high-risk group was the most common (80%). Among the 34 patients who had genotyping performed, the *KIT* exon 11 mutation was the most common (74%), and the *PDGFRA* exon 8 D842V mutation was observed in 3 patients (9%). The pathologic characteristics are summarized in Table 2 and Fig 2.

**Table 1:** Clinical characteristics of patients at surgery (n = 54).

Patient characteristics	Value
Sex, Male	31 (57.4)
Age at surgery, years	
Median, range	60 (33–79)
>60	25 (46.3)
Weight, kg	62.2±8.7
Body mass index, kg/m^2^	23.7±2.7
Diabetes mellitus, present	9 (16.7)
Total cholesterol, mg/dL	168±32
Fasting glucose, mg/dL	112±28
Imatinib therapy	
Pre-operative	2 (3.7)
Post-operative	25 (46.3)

Data are presented as n (%) or mean±SD.

SD = standard deviation.

**Table 2:** Pathological characteristics of gastrointestinal stromal tumors.

Tumor characteristics	n (%)
Tumor status	
Non-metastatic	44 (81.5)
Resectable metastatic	10 (18.5)
Primary site	
Stomach	36 (66.7)
Small bowel	14 (25.9)
Others	4 (7.4)
Histology	
Spindle	35 (64.8)
Epithelioid	9 (16.7)
Mixed	10 (18.5)
Size (cm)	
Median, range	8 (2–47)
>5 cm	38 (70.4)
>5/50 HPF	36 (66.7)
Modified NIH classification	
Low/Intermediate	11 (20.4)
High	43 (79.6)
KIT (IHC), positive	51 (94.4)
CD34 (HC), positive	40 (80.0)
DOG1 (IHC), positive	14 (87.5)
Genotyping (n = 34)	
Exon 11	25 (73.5)
Exon 9	1 (2.9)
D842V	3 (8.8)
Others	5 (9.3)
Progranulin expression	
High	27 (50.0)
Low	27 (50.0)

HPF = high power fields, IHC = immunohistochemical, NIH = National Institutes of Health.

### Clinicopathological features according to PGRN expression

Twenty-seven patients (50%) had high PGRN-expressing tumors with a staining intensity of 2+ or 3+. There was no significant difference in clinicopathologic factors, including sex, age, diabetes, BMI, cholesterol, primary site, size, mitosis and immunohistochemical staining status between patients with high PGRN-expressing and low PGRN-expressing GISTs (Table 3). However, the patients with high PGRN-expressing tumors showed more epithelioid or mixed histology (48% vs. 22%; P = 0.046) and *KIT* exon 11 mutations (91% vs. 46%; P = 0.037) than those with low PGRN-expressing tumors.

**Table 3:** Characteristics according to progranulin expression.

Characteristics	Low PGRN	High PGRN	P-value
Sex			0.409
Male	14 (51.9)	17 (63.0)	
Female	13 (48.1)	10 (37.0)	
Age at surgery			
Median, range	62 (33–79)	58 (35–76)	0.400
>60	14 (51.9)	11 (40.7)	0.413
Diabetes mellitus, present	4 (14.8)	5 (18.5)	0.715
BMI, >25 kg/m^2^	7 (25.9)	6 (22.2)	0.750
Total cholesterol, >200 mg/dL	6 (22.2)	4 (14.8)	0.484
Fasting glucose, >100 mg/dL	17 (63.0)	15 (55.6)	0.580
Primary site			0.564
Stomach	17 (63.0)	19 (70.4)	
Non-stomach	10 (37.0)	8 (29.6)	
Histology			0.046
Spindle	21 (77.8)	14 (51.9)	
Epithelioid/mixed	6 (22.2)	13 (48.1)	
Size (cm)			
Median, range	8 (2–23)	7.5 (3–47)	0.759
>5 cm	18 (66.7)	20 (74.1)	0.551
Mitosis, >5/50 HPF	17 (63.0)	19 (70.4)	0.564
Initial status, metastatic	4 (14.8)	6 (22.2)	0.484
Modified NIH classification			0.735
Low/Intermediate	5 (18.5)	6 (22.2)	
High	22 (81.5)	21(77.8)	
KIT, positive	25 (92.6)	26 (96.3)	1.000
CD34, positive	22 (84.6)	18 (75.0)	0.490
DOG1, positive	8 (100.0)	6 (75.0)	0.467
Genotyping (n = 34)			0.037
Exon 11	6 (46.2)	19 (90.5)	
Exon 9	1 (7.7)	0 (0.0)	
D842V	2 (15.4)	1 (4.8)	
Others	4 (30.8)	1 (4.8)	
Death	1 (3.7)	3 (11.1)	0.299

BMI = body mass index, HPF = high power fields, NIH = National Institutes of Health, PGRN = progranulin.

### Survival outcomes according to PGRN expression

**Fig 3:**
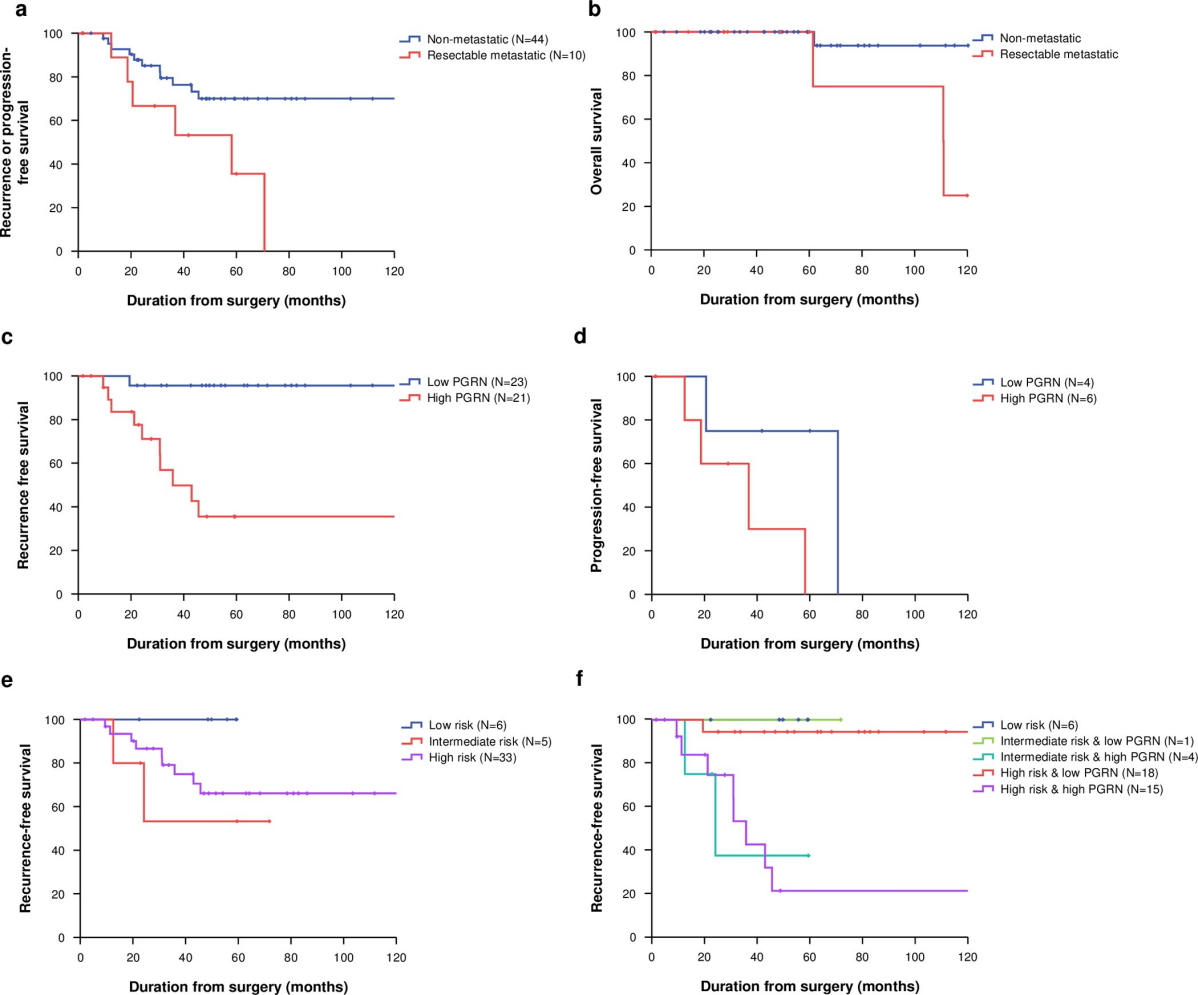
Recurrence or progression-free survival (A) and overall survival (B) according to initial status; recurrence-free survival (C) in non-metastatic gastrointestinal stromal tumors (GISTs) and progression-free survival (D) in metastatic GISTs according to progranulin (PGRN) expression; recurrence-free survival in non-metastatic GISTs according to modified National Institutes of Health (NIH) classification (E) and modified NIH/PGRN expression (F).

The median follow-up duration of 50 living patients was 52.8 months (interquartile range [IQR], 27.7–73.5). Most death events were observed in metastatic tumors (N = 3, 75%) and in high PGRN-expressing tumors (N = 3, 75%). According to tumor status, the 5-year RFS of non-metastatic and metastatic patients was 70.0% (95% CI 56.5–83.5) and 35.6% (95% CI 6.0–65.3), respectively (P = 0.023; Fig 3). In the patients with non-metastatic GISTs, the 5-year RFS of low-risk, intermediate-risk, and high-risk patients according to the modified NIH classification was 100.0%, 53.3% (95% CI 10.0–97.0), and 66.1% (95% CI 50.0–82.3), respectively (P = 0.242). Patients with high PGRN-expressing tumors had a worse 5-year RFS (35.6%, 95% CI 15.1–56.1) compared to those with low PGRN-expressing tumors (95.7%, 95% CI 87.4–100.0, P<0.001; Table 4). Furthermore, the non-metastatic patients were categorized into five groups by combining PGRN expression and the modified NIH classification; the 5-year RFS was evaluated as follows: 1) low risk (regardless of PGRN) = 100.0%; 2) intermediate risk with low PGRN = 100.0%; 3) intermediate risk with high PGRN = 37.5% (95% CI 0.0–84.9); 4) high risk with low PGRN = 94.4% (95% CI 83.8–100.0); and 5) high risk with high PGRN = 21.3% (95% CI 1.0–42.0, P<0.001). In the patients with metastatic GISTs, there was a trend of poor RFS in the high PGRN-expressing tumors (median, 36.8 months, 95% CI 8.6–65.0) compared to the low PGRN-expressing tumors (70.7 months, 95% CI not evaluable; P = 0.104). The 5-year OS of non-metastatic and metastatic patients was 93.8% (95% CI 86.7–100.0) and 75.0% (95% CI 48.2–100.0), respectively (P = 0.021). Multivariate analysis in non-metastatic disease showed that high PGRN expression (hazard ratio [HR] 51.4, 95% CI 3.2–821.9, P = 0.005) and old age (>60 years; HR 5.7, 95% CI 1.01–31.7, P = 0.049) were independent prognostic factors associated with poor RFS (Table 4).

**Table 4:** Univariate and multivariate analysis for recurrence-free survival (RFS) in non-metastatic disease (n = 44).

Characteristics	5Y-RFS (95% CI)	P-value	HR (95% CI)	P-value
Sex		0.482		0.260
Male	64.8 (46.4–83.2)		Reference	
Female	76.5 (56.9–96.1)		3.22 (0.42–54.54)	
Age, years		0.077		0.049
>60	83.3 (67.7–98.9)		Reference	
≤60	56.8 (36.1–77.5)		5.66 (1.01–31.65)	
Primary site		0.788		0.095
Stomach	70.9 (55.2–86.6)		Reference	
Non-gastric	66.7 (40.0–93.4)		13.54 (0.64–288.77)	
Histology		0.705		0.191
Epithelioid/mixed	62.5 (37.1–87.9)		Reference	
Spindle	73.3 (57.5–89.1)		4.76 (0.46–49.21)	
Size, cm		0.608		0.095
≤5	76.2 (53.1–99.3)		Reference	
>5	66.9 (50.3–83.5)		5.57 (0.74–42.03)	
Mitosis		0.056		0.122
≤5/50 HPF	91.7 (78.2–100.0)		Reference	
>5/50 HPF	59.2 (41.0–77.4)		11.79 (0.52–268.78)	
Modified NIH classification		0.521		
Low/Intermediate	79.5 (55.6–100.0)			
High	66.1 (49.9–82.3)			
Genotyping (n = 25)		0.548		
Exon 11	44.6 (21.6–67.6)			
Others	71.4 (37.9–100.0)			
Post-operative imatinib		0.750		0.667
Not received	67.2 (50.1–84.3)		Reference	
Received	77.5 (56.4–98.6)		1.55 (0.21–11.39)	
Progranulin expression		<0.001		0.005
Low	95.7 (87.4–100.0)		Reference	
High	35.6 (15.1–56.1)		51.43 (3.22–821.96)	

5Y-RFS = 5-year recurrence-free survival, CI = confidence interval, HR = hazard ratio, HPF = high power fields, NIH = National Institutes of Health.

## Discussion

This study investigated the PGRN expression in resected GISTs and evaluated the clinicopathologic features of patients with GISTs according to PGRN expression. High PGRN-expressing tumors showed more epithelioid type histology and exon 11 mutation tumors. The RFS was worse in patients with GISTs with high PGRN expression regardless of the modified NIH risk assessment.

### Clinicopathological features of PGRN-expressing GISTs

In several tumors, the relationships between PGRN expression and prognosis have been reported. Recently, PGRN has been evaluated as an adipose tissue hormone (adipokine) implicated in obesity and insulin resistance [11]. Particularly, PGRN indicated a poor prognosis in tumors mostly associated with metabolic syndrome such as breast cancer [10, 12], colorectal cancer [13], gallbladder cancer [9], bladder cancer [14], and prostate cancer [15]. In practice, patients with GISTs are usually diagnosed without symptoms, even in the advanced stage. Therefore, we evaluated the association between PGRN expression and metabolic parameters, but no association was observed with diabetes mellitus, body weight, BMI, cholesterol level, and fasting glucose levels. It is noteworthy, however, that high PGRN-expressing tumors show increased epithelioid/mixed type histology and *KIT* exon 11 mutations because several previous studies have shown that non-spindle cell histology and exon 11 mutations are associated with poor prognosis for GIST [16–18].

### Clinical impact of PGRN-expressing GISTs

In multivariate analysis for RFS, old age and PGRN expression were found to be significant predictors of poor prognosis. As mentioned above, there was no significant difference in RFS according to risk classification, including tumor size and mitosis index. However, when PGRN expression and risk group classification were combined, intermediate-risk patients with high PGRN-expressing tumors, for whom postoperative imatinib was not recommended, showed worse prognosis than high-risk patients with low PGRN-expressing tumors, although imatinib was administered to some patients. This suggested that PGRN overexpression was significantly associated with poor RFS in patients with GISTs who had undergone curative resection. Furthermore, there was a trend of poor RFS in high PGRN-expressing tumors among the patients with resectable metastatic GISTs. Currently, however, there is limited understanding of the role of PGRN in GISTs. PGRN is known to promote proliferation and angiogenesis through TNFR2/Akt and ERK signaling pathways in other gastrointestinal tumors [8], and further investigation is needed to discover the role of PGRN as a biomarker and therapeutic target in GISTs.

### Postoperative imatinib therapy during study periods

Recently, postoperative treatment has been standardized as a 3-year use of the adjuvant imatinib in patients with high-risk GISTs, based on a large phase III trial in which imatinib use for 36 months reduced the risk of recurrence and improved overall survival (OS) compared with imatinib use for 12 months in patients with GISTs with a high risk of recurrence after surgery [4, 5]. In this study, 43 patients were at high risk after surgery, and 25 (58%) received postoperative adjuvant imatinib therapy. Because of the introduction of postoperative imatinib therapy, it seems that there was no significant difference in RFS in the high-risk group and low- or intermediate-risk group according to the modified NIH risk. In Korea, the adjuvant imatinib therapy for 1 year was reimbursed from 2010, and adjuvant treatment for 3 years could become a standard therapy since the reimbursement eventually expanded to 3 years in November 2013. Imatinib was heterogeneously administered because this study included patients who had undergone surgical treatment for 11 years from 2007 to 2017.

### Limitations and conclusions

Although the PGRN expression status was a statistically significant factor in terms of prediction of poor RFS, the results should be interpreted with caution. This study might be biased by the small size of cohort and retrospective design in a single institution; therefore, further studies are needed to confirm our results.

In conclusion, high PGRN-expressing GISTs had more epithelioid/mixed histology and *KIT* exon 11 mutations. Patients with GISTs expressing high levels of PGRN had significantly poorer outcomes than patients whose tumors expressed low levels of PGRN. Therefore, PGRN expression status may be a potential prognostic factor for patients with GISTs who have undergone curative resection.
